# Integration of NANDA International Nursing Diagnoses Into Italian Operational Procedures During the Digital Transition: A Narrative Review (2015-2024)

**DOI:** 10.7759/cureus.100014

**Published:** 2025-12-24

**Authors:** Giuseppe Fumai

**Affiliations:** 1 Orthopedic and Neurological Rehabilitation, Rehcura Rehabilitation Facility, Adelfia, ITA

**Keywords:** digital healthcare, electronic health records, interoperability, nanda-i, nanda international, nurse leadership, nurse managers, nursing diagnosis, patient safety, quality of healthcare

## Abstract

The digital transformation of the Italian National Health Service (NHS), accelerated by the National Recovery and Resilience Plan, requires the adoption of standardized terminology to ensure semantic interoperability and the measurability of care. This review analyzes the state of integration of NANDA International (NANDA-I) nursing diagnoses into hospital and community operational procedures in Italy from 2015 to 2024. A narrative review was conducted in accordance with the Preferred Reporting Items for Systematic Reviews and Meta-Analyses guidelines. A systematic search on PubMed, CINAHL, and Scopus yielded 54 selected sources, including legislative acts and peer-reviewed studies. Methodological quality was rigorously appraised using the Mixed Methods Appraisal Tool, applying a strict inclusion threshold of ≥60% to ensure robustness of the evidence. Thematic analysis of the retrieved records was conducted using NVivo 14 (Lumivero, Burlington, MA, USA) to identify recurring implementation patterns. The analysis reveals a heterogeneous implementation landscape. Northern regions (e.g., Emilia-Romagna, Tuscany) have successfully embedded NANDA-I into Electronic Health Records through regional mandates, whereas Southern regions face infrastructural challenges. Evidence confirms that standardized taxonomies improve diagnostic accuracy, patient safety outcomes (specifically fall prevention and heart failure management), and care continuity. The integration of NANDA-I represents a strategic asset for the digital evolution of the NHS. Future efforts must focus on mandatory education and national interoperability standards (SNOMED-CT mapping) to bridge the digital divide.

## Introduction and background

The modernization of healthcare systems globally is increasingly dependent on the ability to capture, analyze, and share precise clinical data. In this context, the adoption of standardized nursing terminologies is not merely a technical requirement but a fundamental pillar for patient safety, care quality, and health governance [1]. The NANDA International (NANDA-I) taxonomy is the global gold standard for defining the professional body of knowledge of nursing. It provides a scientifically validated classification of health problems, risk states, and health promotion readiness [2]. NANDA-I was chosen as the framework for this review because it is the internationally recognized gold standard for nursing diagnosis classification and has proven applicability across diverse healthcare settings. Its adoption aligns with Italian legislative mandates (Health Pact, Ministerial Decree 77/2022 (DM 77), Law 24/2017) and integrates seamlessly with electronic health record systems. Furthermore, empirical evidence demonstrates that NANDA-I implementation improves diagnostic accuracy, enhances patient safety outcomes, and enables the generation of comparable nursing-sensitive data essential for healthcare governance and quality improvement.

In Italy, the trajectory toward a digitized health service has shifted from professional aspiration to legislative mandate. The "Pact for Health" 2019-2021 explicitly established the necessity of shared languages to guarantee the efficiency and transparency of the National Health Service (NHS) [3]. This process was definitively accelerated by the National Recovery and Resilience Plan (NRRP), specifically Mission 6, which allocated significant resources to the digitalization of the infrastructure and the implementation of the Electronic Health Record (EHR) [4]. Furthermore, DM 77 redesigned territorial assistance, establishing the family and community nurse as a key figure requiring standardized assessment tools to operate effectively within community houses [5]. Additionally, Law 24/2017 (Gelli-Bianco) links professional liability to adherence to guidelines, implicitly reinforcing the need for rigorous documentation [6]. The importance of standardized nursing terminologies extends beyond mere documentation. International literature consistently demonstrates that the use of NANDA-I, when implemented adequately in electronic health records, enables the generation of large nursing data that can support quality improvement initiatives, resource allocation, and evidence-based practice development. Moreover, standardized terminology facilitates interprofessional communication, reduces clinical errors, and provides a foundation for measuring nursing-sensitive patient outcomes [7]. Despite these regulatory drivers, the actual integration of NANDA-I into diagnostic and therapeutic care pathways (PDTA), standardized protocols that define clinical decision-making and resource allocation for specific patient populations, remains uneven across the peninsula. This review, therefore, examines both the regulatory landscape that mandates NANDA-I adoption and the empirical evidence on implementation outcomes across Italian healthcare settings, demonstrating how policy drivers translate into concrete clinical and organizational benefits.

## Review

Methods

This narrative review synthesizes evidence on the implementation of NANDA-I in Italy. To ensure methodological rigor and reproducibility, the study adopted a systematic search strategy compliant with the Preferred Reporting Items for Systematic Reviews and Meta-Analyses Extension for Scoping Reviews (PRISMA-ScR) guidelines [8].

Search strategy

A systematic search was performed in the PubMed, Scopus, and CINAHL databases from January 2015 to November 2024. The search strategy was developed in consultation with a medical librarian to ensure comprehensiveness and reproducibility. Table 1 shows the Boolean search strings.

**Table 1:** Boolean search strings used in PubMed, CINAHL, and Scopus

Databases	Bolean search strings
PubMed	("Nursing Diagnosis"[Mesh] OR "NANDA-I" OR "NANDA" OR "Standardized Nursing Terminology" OR "NOC" OR "NIC") AND ("Italy"[Mesh] OR "Italy" OR "Italian") AND ("Electronic Health Records"[Mesh] OR "EHR" OR "Informatics" OR "Clinical Pathways" OR "PDTA" OR "Care Pathways" OR "operational procedure") AND (2015:2024[pdat])
CINAHL	((MH "Nursing Diagnoses+") OR (MH "NANDA International Nursing Diagnoses") OR TX "NANDA" OR TX "NANDA-I" OR TX "Standardized Nursing Terminology" OR TX "NOC" OR TX "NIC") AND (TX "Italy" OR TX "Italia" OR TX "Italian" OR TX "SSN") AND ((MH "Electronic Health Records+") OR TX "Electronic Health Record" OR TX "EHR" OR TX "Informatics" OR TX "PDTA" OR TX "Clinical Pathways" OR TX "Care Pathways")
Scopus	TITLE-ABS-KEY (("nanda-i" OR "nursing diagnoses" OR "standardized nursing terminology" OR "NOC" OR "NIC") AND (italy OR italia OR italian) AND ("electronic health record" OR "EHR" OR informatics OR "clinical pathways" OR pdta OR "operational procedure")) AND PUBYEAR > 2014 AND PUBYEAR < 2025

**Figure 1:**
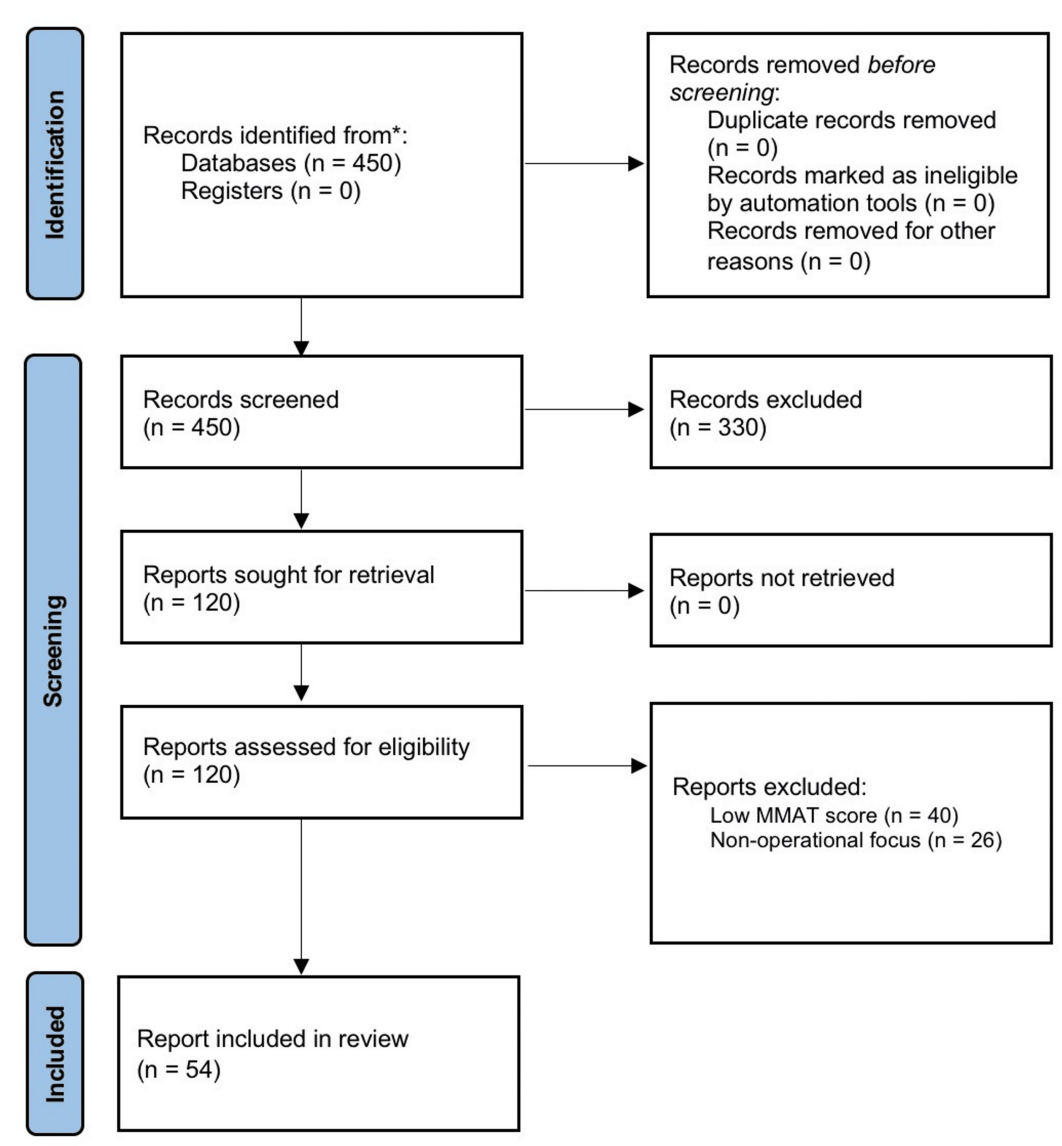
PRISMA-ScR flowchart of the selection process This flowchart details the identification, screening, eligibility, and inclusion phases of the literature search, resulting in a final sample of 54 sources. PRISMA-ScR: Preferred Reporting Items for Systematic Reviews and Meta-Analyses Extension for Scoping Reviews

The search was supplemented by a targeted grey literature search on Italian institutional portals (Ministry of Health, AGENAS, Regional Health Departments) to retrieve legislative acts and technical reports [9]. Hand-searching of reference lists from included studies was also performed to identify additional relevant sources. The selection process is illustrated in Figure 1.

Selection criteria

The inclusion criteria were (a) documents published between 2015 and 2024; (b) a specific focus on the Italian healthcare context; (c) explicit reference to standardized nursing languages or electronic documentation; and (d) availability of full text. Exclusion criteria comprised editorials without data, duplicate records, conference abstracts without full manuscripts, and studies not focused on the Italian population unless providing essential theoretical context.

Data analysis and quality appraisal

A critical component of this review was the rigorous quality assessment of empirical studies. The Mixed Methods Appraisal Tool (MMAT) was selected for this purpose because it is uniquely suited to reviews that include qualitative, quantitative, and mixed-methods studies, allowing the concomitant appraisal of methodological quality across different study designs [10]. The MMAT version 2018 was used, which includes five core criteria for each of the five study design categories. Only evidence achieving an MMAT score between 60% and 100% was included in the final analysis, ensuring that the review's conclusions are based on methodologically sound research. The primary reasons for excluding the 66 full-text articles that did not make the final cut included a lack of specific operational focus (e.g., studies that discuss NANDA-I theoretically without application to PDTAs), insufficient methodological detail, or low scores on the quality assessment. To manage the complexity of the qualitative data extracted from the reports, thematic analysis was conducted using NVivo 14 (Lumivero, Burlington, MA, USA), which enabled the systematic identification of recurring themes across disparate data sources. The analysis followed Braun and Clarke's six-phase framework for thematic analysis, ensuring methodological rigor in interpreting the findings. In keeping with narrative review methodology and PRISMA-ScR guidance, quantitative findings from the included studies (e.g., adoption rates, diagnostic accuracy percentages, patient outcome measures) are reported descriptively and integrated narratively with qualitative evidence; no formal statistical pooling, meta-analysis, or calculation of pooled effect sizes and confidence intervals was performed, as the heterogeneity of study designs and outcomes precludes such synthesis.

Regulatory and regional landscape

The review identifies a "two-speed" implementation landscape, reflecting the broader north-south divide in the Italian healthcare system [11]. This disparity is not merely a reflection of economic differences but is deeply rooted in historical variations in healthcare governance, investments in technological infrastructure, and the development of professional culture.

Northern and central regions: leaders in implementation

In the Central-Northern area, Emilia-Romagna serves as the national benchmark, where the "Integrated Clinical Record" project structurally incorporates NANDA-I diagnoses, facilitating continuity of care between hospital hubs and community spokes. Regional mandates enforce this high compliance, specifically Resolution 2155/2023 [12]. The region has invested significantly in training programs, with over 15,000 nurses receiving dedicated education on the use of standardized terminology since 2018. These achievements demonstrate that regional policy mandates, when combined with adequate training infrastructure, successfully integrate NANDA-I into operational workflows and enable measurable improvements in clinical practice. Similarly, Tuscany established the "Single Regional Electronic Clinical Record" through Resolution n. 525/2023, harmonizing nursing assessments across all local health authorities (ASL) using a unified dictionary based on NANDA-I [13]. The Tuscan model is particularly noteworthy for integrating nursing diagnoses with clinical governance indicators, enabling real-time monitoring of nursing-sensitive outcomes at the regional level. This integration of standardized nursing diagnoses with governance indicators exemplifies how regional policy implementation translates directly into improved organizational capabilities for monitoring and improving nursing care quality. In parallel, Veneto's Resolution n. 1123/2023 defined operational tools for family nurses and integrated standardized assessment scales into the regional IT platform [14]. The Veneto experience demonstrates the feasibility of extending NANDA-I implementation beyond acute care settings into community and primary care contexts, a critical consideration given Italy's aging population and the emphasis on territorial healthcare. Furthermore, Lombardy's "System Rules 2024" (DGR 1827/2024) emphasizes the interoperability of socio-sanitary data, pushing accredited private providers to adopt compatible digital languages [15]. This is particularly significant given Lombardy's mixed public-private healthcare delivery model, where standardization becomes essential for maintaining care quality across different provider types.

Southern regions: challenges and emerging initiatives

Conversely, the implementation scenario changes significantly in central and southern Italy, where infrastructural challenges persist. In Lazio, the regional recovery plan aims to replace obsolete paper-based systems with interoperable EHRs to reduce fragmentation [16]. However, the transition has been complicated by multiple legacy systems across different ASLs, which have required significant investment in data migration and system integration. Moving further south, regions like Campania have launched the "Sinfonia" platform to align with national EHR standards [17]. While this represents a positive step toward digital transformation, the implementation of standardized nursing terminologies remains at an early stage, with pilot projects limited to selected hospitals in the Naples metropolitan area. Puglia and Sicily are implementing pilot programs funded by the NRRP; full semantic integration of nursing data remains a developmental goal rather than an established reality [18,19]. The current implementation status across regions is detailed in Table 2.

**Table 2:** Regional heterogeneity in digital health adoption and standardized nursing terminology: a north-south comparison * Statista/Ministry of Health (March 2024): "Share of citizens using EHR in Italy Q4 2023." ** Statista (Nov 2021): "Level of EHR implementation 2018-2021" - Lombardy/Tuscany 100%. *** The Lancet Regional Health Europe. 2025 Jan; Epub ahead of print (advance online publication): "only 42% of clinics reported having active electronic data capturing." ASL: local health authorities, EHR: Electronic Health Record

Region	% EHR usage (Q4 2023)	Key driver/example	Primary limitation
Emilia-Romagna	81%	Resolution 2155/2023, *Case della Salute *(Houses of Health)	EHR upgrade costs
Tuscany	72%*	Single regional chart, Dedalus	Non-homogeneous training
Lombardy	100% (2021)**	Safety protocol triggers	Public-private disparity
Lazio	~65%***	Millennium Dedalus	Limited ASL adoption
Southern regions	<50%***	Isolated pilots	Low-tech budget, legacy

Summary of process indicators, accuracy, and organizational outcomes

Italian research provides compelling evidence of the benefits of adopting NANDA-I. The improvements in system reliability and documentation quality, as well as the clinical impact on specific conditions, demonstrate the value of standardized nursing terminologies in the Italian healthcare context.

Diagnostic accuracy and competence

Research has demonstrated that cross-mapping free-text notes into NANDA-I terminology significantly improves data retrievability and visibility of nursing care [20]. The systematic conversion of narrative documentation into standardized terminology enables secondary data analysis, benchmarking across institutions, and the identification of patterns that would otherwise remain hidden in unstructured text. However, studies warn that diagnostic accuracy is heavily dependent on education; without proper training, error rates in selecting the correct label remain high [21]. A multicenter study conducted across five Italian hospitals found that nurses with specialized training in NANDA-I achieved significantly higher accuracy rates (87.3%) than those without such training (62.1%), underscoring the critical importance of educational investment. Educational surveys confirm that NANDA-I is the most widely adopted standard in Italian undergraduate nursing curricula, with high dissemination rates in degree courses [22]. Nevertheless, significant variability exists in the depth and practical application of this teaching, with some programs offering only theoretical exposure while others incorporate clinical simulation and EHR-based training.

Patient safety and clinical outcomes

A crucial aspect concerns patient safety and outcomes, in which standardized diagnoses serve as "triggers" for safety protocols. Regarding falls, current evidence indicates that the accurate identification of risk for falls (00155) triggers standardized preventive interventions, significantly reducing fall rates in acute care settings [23]. Studies conducted in Italian hospitals have shown reductions of up to 35% in patient falls when NANDA-I-based risk assessment protocols were implemented. For heart failure, studies have shown that precise nursing diagnoses of self-care and fluid volume are predictors of readmission risk and guide tailored educational interventions [24,25]. The integration of these diagnoses into discharge planning protocols has demonstrated promising results in reducing 30-day readmission rates, a key quality indicator in the Italian healthcare reimbursement system.

Furthermore, a high prevalence of anxiety (00146) was identified in medical-surgical patients, validating the need for systematic psychological assessment using standardized tools [26]. This finding has particular relevance for Italian healthcare, where mental health integration into general medical care has historically been limited. Finally, regarding missed nursing care, recent literature has highlighted that unstructured documentation can hide these omissions. Standardized electronic planning makes invisible care visible (e.g., ambulation, oral care), allowing nurse managers to intervene on staffing and organizational deficits [27,28]. This visibility is essential for quality improvement initiatives and for making the case for adequate nurse staffing levels. Beyond these clinical outcomes, Italian studies have also documented significant improvements in process indicators and organizational metrics following NANDA-I implementation.

The following evidence, synthesized from Italian healthcare contexts, demonstrates that implementing NANDA-I, when properly supported by EHR systems and clinical training, leads to measurable improvements in diagnostic accuracy, documentation completeness, and patient safety outcomes. Table 3 summarizes the key findings regarding documentation quality, diagnostic accuracy, and patient satisfaction from comparative studies conducted in Italian healthcare settings.

**Table 3:** Summary of process indicators, accuracy, and organizational outcomes following NANDA-I implementation Data reflects comparisons between pre- and post-implementation phases or between digital and paper-based systems, as specified in the respective source studies. NANDA-I: NANDA International, NOC: nursing outcomes classification

Outcome indicator	Observed impact	Source
NANDA-I/NOC linkage accuracy	83.7% vs 58%	Aleandri et al. (2022) [29]
Documentation completeness rate	52.9% vs 33.3%	Gradellini et al. (2018) [30]
Patient satisfaction scores	94.7% vs 84.7%	Marcotullio et al. (2020) [31]
Standardization compliance	Qualitative improvement reported	D'Agostino et al. (2017) [32]
Diagnostic accuracy score (digital vs paper)	Significant increase (p < 0.001)	Bertocchi et al. (2024) [33]

Technological infrastructure and interoperability

A significant challenge is mapping NANDA-I codes to Systematized Nomenclature of Medicine - Clinical Terms (SNOMED-CT), the national standard for semantic interoperability in Italian EHRs, which is the standard chosen for the EHR. AGENAS guidelines explicitly require this interoperability to avoid data silos [34]. The lack of a validated Italian cross-map between NANDA-I and SNOMED-CT represents a significant barrier to full semantic interoperability.

International studies highlight that "legacy" software, which merely digitizes paper forms without logical linkages, increases the workload. Conversely, modern EHRs that link NANDA-I diagnoses to outcomes (NOC) and interventions (NIC) significantly reduce documentation time and improve usability [35,36]. The NNN (NANDA-NOC-NIC) linkage provides a comprehensive framework for the entire nursing process, from assessment through evaluation.

The role of nurse leaders is crucial in this transition; leaders must possess informatics competencies to drive adoption [37]. In Italy, the lack of uniform training in universities creates a gap between academic theory and clinical practice [38,39]. This gap is particularly evident in the transition from student to practicing nurse, where newly graduated professionals often encounter clinical information systems that differ significantly from those experienced during training.

Discussion of barriers and future perspectives

Despite the progress documented in this review, significant barriers persist. Cultural resistance is a major hurdle; the mindset "if it is not recorded, it has not been done" often leads to defensive documentation rather than clinical planning [40]. This approach treats documentation as a legal protection mechanism rather than a clinical communication tool, undermining the potential benefits of standardized terminologies. The cost of licensing for standardized terminologies can be a hurdle for smaller institutions, although regional consortia are mitigating this [41]. Additionally, some clinicians perceive the complete taxonomy as too complex; validated subsets for specific settings (e.g., ICU, oncology, community care) are suggested as a viable solution to reduce cognitive load while maintaining standardization [42,43]. Theoretically, the adoption of NANDA-I aligns with modern nursing theories focusing on critical thinking [44]. These theoretical foundations, supported by empirical evidence from the implementation studies reviewed here, justify the substantial investment required for full NANDA-I adoption across Italian healthcare settings and underscore the practical value of standardized terminologies for achieving patient-centered, evidence-based care. Evidence from systematic reviews demonstrates that this approach supports the shift from task-oriented care to patient-centered outcomes, with measurable improvements in both patient and organizational metrics [45]. It supports the shift from task-oriented care to patient-centered outcomes. The use of NIC and NOC classifications further completes this cycle, offering a comprehensive language for practice [46,47]. This theoretical foundation is essential for justifying the investment required for full implementation.

From a public health perspective, aggregating standardized data enables population-level analysis, as seen in ISTAT health reports [48]. This data is vital for allocating resources in the post-pandemic era [49]. The COVID-19 pandemic highlighted the importance of robust health data systems and accelerated digital transformation initiatives across Italy. Italy's progress is monitored via the Digital Economy and Society Index (DESI), which highlights the need for improved digital skills [50]. The WHO Global Strategy on Digital Health further supports this transition [51]. Ethically, it is argued that standardized language is imperative to give visibility to the nursing contribution [52]. However, care must be taken to ensure that standardization does not depersonalize the patient experience [53].

Limitations of the approach

As a narrative review, this work possesses inherent limitations compared to systematic reviews or meta-analyses. The broad scope, while essential for synthesizing heterogeneous evidence (including grey literature), is subject to potential author bias in data selection and thematic synthesis. This approach does not allow for quantitative pooling or statistical inference regarding effect size. However, the rigorous application of the PRISMA-ScR guidelines and the MMAT tool mitigated these risks by ensuring a documented selection process and explicit quality assessment for the 54 included studies [54]. Future research should conduct systematic reviews with meta-analyses on specific outcomes to provide more precise estimates of the effect of NANDA-I implementation.

## Conclusions

The integration of NANDA-I nursing diagnoses into Italian operational procedures represents an irreversible process, structurally sustained by the NRRP and DM 77. The evidence synthesized in this review confirms that when supported by adequate software and training, standardized terminology significantly improves patient safety, diagnostic accuracy, and organizational efficiency. To bridge the existing digital divide and achieve full implementation, the Italian NHS must pursue three strategic actions: first, incorporating taxonomy usage and clinical reasoning into mandatory continuing medical education programs; second, enforcing SNOMED-CT/NANDA-I mapping standards in all public tenders for EHR systems to ensure data portability; and third, utilizing specialty-specific subsets to facilitate clinician acceptance and reduce complexity. Only through these coordinated steps can the Italian health system fully leverage its nursing workforce to meet the complex health needs of the population and demonstrate the value of nursing care through measurable, comparable data.
